# Targeting TRPM2 Channels Impairs Radiation-Induced Cell Cycle Arrest and Fosters Cell Death of T Cell Leukemia Cells in a Bcl-2-Dependent Manner

**DOI:** 10.1155/2016/8026702

**Published:** 2015-12-29

**Authors:** Dominik Klumpp, Milan Misovic, Kalina Szteyn, Ekaterina Shumilina, Justine Rudner, Stephan M. Huber

**Affiliations:** ^1^Department of Radiation Oncology, University of Tübingen, 72076 Tübingen, Germany; ^2^Department of Physiology, University of Tübingen, 72076 Tübingen, Germany; ^3^Department of Oral & Maxillofacial Surgery, The University of Texas Health Science Center, San Antonio, TX 78229, USA; ^4^Institute for Cell Biology (Cancer Research), University Hospital Essen, University of Duisburg-Essen, 45122 Essen, Germany

## Abstract

Messenger RNA data of lymphohematopoietic cancer lines suggest a correlation between expression of the cation channel TRPM2 and the antiapoptotic protein Bcl-2. The latter is overexpressed in various tumor entities and mediates therapy resistance. Here, we analyzed the crosstalk between Bcl-2 and TRPM2 channels in T cell leukemia cells during oxidative stress as conferred by ionizing radiation (IR). To this end, the effects of TRPM2 inhibition or knock-down on plasma membrane currents, Ca^2+^ signaling, mitochondrial superoxide anion formation, and cell cycle progression were compared between irradiated (0–10 Gy) Bcl-2-overexpressing and empty vector-transfected Jurkat cells. As a result, IR stimulated a TRPM2-mediated Ca^2+^-entry, which was higher in Bcl-2-overexpressing than in control cells and which contributed to IR-induced G_2_/M cell cycle arrest. TRPM2 inhibition induced a release from G_2_/M arrest resulting in cell death. Collectively, this data suggests a pivotal function of TRPM2 in the DNA damage response of T cell leukemia cells. Apoptosis-resistant Bcl-2-overexpressing cells even can afford higher TRPM2 activity without risking a hazardous Ca^2+^-overload-induced mitochondrial superoxide anion formation.

## 1. Introduction

*Transient Receptor Potential (TRP) Cation Channels*. The TRP superfamily comprises a diverse range of Ca^2+^-permeable cation channels [1]. TRP channels contribute to changes in cytosolic free Ca^2+^ (_free_[Ca^2+^]_i_) by directly acting as Ca^2+^ entry channels in the plasma membrane or by changing membrane potentials, modulating the activity and/or driving forces for the Ca^2+^ entry channels [2]. The melastatin subfamily (TRPM) has been subdivided into three subgroups on the basis of sequence homology (TRPM1/TRPM3, TRPM4/TRPM5, and TRPM6/7) with TRPM8 and TRPM2 being distinct proteins [3]. The Ca^2+^-permeable TRPM2 channels, formerly known as TRPC2 and LTRPC2, were first identified in 1998 [4]. Reactive oxygen species (ROS) have been demonstrated to induce TRPM2 currents and increase _free_[Ca^2+^]_i_ in various cell types transfected with TRPM2 [5], as well as in pancreatic *β*-cells [6], neutrophil granulocytes [7], and U937 monocytes [8].

*TRPM2 and Cell Death*. By increasing _free_[Ca^2+^]_i_, TRPM2 may increase the susceptibility to cell death suggesting that TRPM2 channels function as “death channels.” As a matter of fact, heterologous expression of TRPM2 in human embryonic kidney cells [9] or A172 human glioblastoma cells [10] facilitates oxidative stress-induced cell death. Moreover, expression of TRPM2 has been demonstrated in several tumor entities such as insulinoma [6], hepatocellular carcinoma [6], prostate cancer [11], lymphoma [12], leukemia [13], and lung cancer cell lines [14] in which TRPM2 reportedly may foster cell death [15].

*Ca* ^*2+*^ *-Signaling by TRPM2*,* Bcl-2, and Mitochondria*. ROS-induced TRPM2 channel activation most probably occurs indirectly via formation of adenosine diphosphate ribose (ADPR) which activates the channel by binding to a special domain located at the C-terminus of the channel [16]. ADP-ribose polymers are formed during DNA damage response by poly(ADP-ribose) polymerases (PARPs). Upon DNA repair ADPR is released from the ADPR polymers by glycohydrolases [17, 18]. Another main source of ADPR is the mitochondria [19].

Mitochondrial Ca^2+^ absorbance exerts Ca^2+^ buffering function (for review see [20]). The mitochondrial respiratory chain and the mitochondrial permeability transition pore (PTP) are regulated by Ca^2+^. Moderate mitochondrial Ca^2+^ increase may disinhibit the respiratory chain leading to ΔΨ_*m*_ hyperpolarisation [21] which in turn is accompanied by increasing superoxide anion formation [22]. Mitochondrial Ca^2+^ overload, in contrast, opens the PTP leading to ΔΨ_*m*_ dissipation, cytochrome C release, and apoptotic cell death [20].

The antiapoptotic protein Bcl-2 is a key player in cellular Ca^2+^ homeostasis. In some cell models, overexpression of Bcl-2 reportedly may increase the Ca^2+^ leakage through IP_3_ receptor subtypes in the ER membrane and decrease the ER Ca^2+^ filling. More recent studies, in contrast, suggest an inhibition of IP_3_-receptor-mediated Ca^2+^ release by Bcl-2. Like Bcl-2-caused Ca^2+^ store depletion, Bcl-2-mediated IP_3_-receptor inhibition is thought to prevent proapoptotic bulk Ca^2+^ release from the ER (for review see [23–26]).

*Direct and Indirect Oxidative Stress Conferred by Ionizing Radiation*. Most energy of ionizing radiation (IR) is absorbed by cell water leading to formation of hydroxyl radicals (for review see [27]). Oxidative stress- and DNA repair-associated release of ADP-ribose is supposed to increase the plasma membrane Ca^2+^ permeability by activating TRPM2 channels. Subsequent changes in _free_[Ca^2+^]_i_ and mitochondrial function are under the control of Bcl-2. Together, this hints to a crosstalk between Ca^2+^ and ROS signaling involving TRPM2 Ca^2+^-permeable channels in the plasma membrane, the Ca^2+^-regulated ΔΨ_*m*_ across the inner mitochondrial membrane, and the antiapoptotic protein Bcl-2 in the ER and outer mitochondrial membrane of irradiated cells.

*Aim of the Study*. The present study aimed to define this crosstalk in human T cell leukemia cells subjected to ionizing radiation. To this end, Jurkat cells stably transfected with Bcl-2 or the empty control vector were irradiated with 0, 5, or 10 Gy by 6 MV photons. Ion channel activity, Ca^2+^ signaling, mitochondrial superoxide anion formation, cell cycle control, and cell death were assessed by patch-clamp whole-cell recording, fura-2 Ca^2+^ imaging, immunoblotting, and flow cytometry in irradiated and nonirradiated cells, respectively. In addition, mRNA data of hematopoietic and lymphoid tissue cancer cell lines of the Novartis and Broad Institute Cancer Cell Line Encyclopedia were queried for TRPM2 and Bcl-2 mRNA abundance.

## 2. Material and Methods

### 2.1. Cell Culture

Jurkat E6.1 T cell leukemia cells were from ATCC (Bethesda, Maryland, USA). Jurkat cells stably expressing Bcl-2 (Jurkat-Bcl-2) or a control vector (Jurkat-vector) were prepared as described before [28, 29]. Inducible Bcl-2 transfectants were generated as described [30]. To suppress Bcl-2 expression in Tet-off Jurkat cells, Bcl-2 transfectants were treated with 1 *μ*g/mL doxycycline (Clontech, Heidelberg, Germany) for 48 h. As control cells, the maternal Jurkat Tet-off cells were used. Cells were grown in RPMI 1640 medium supplemented with 10% fetal calf serum (Gibco Life Technologies, Eggenstein, Germany) and maintained in a humidified incubator at 37°C and 5% CO_2_.

### 2.2. Transfection with siRNA

Transfection with siRNA was performed as described [31]. In brief, cells were cultured at a low density to ensure log phase growth. For transfection, 2 × 10^6^ cells were resuspended in 200 *μ*L RPMI 1640 without phenol red. Shortly before transfection, TRPM2 or nontargeting siRNA was added at a concentration of 1 *μ*M. TRPM2 ON-TARGET SMARTpool and the siCONTROL NON-TARGETING pool siRNA were purchased from Dharmacon (Chicago, IL, USA). Cells were electroporated in a 4 mm cuvette in an EPI2500 electroporator (Fischer, Heidelberg, Germany) at 370 V for 10 ms. Immediately after transfection, cells were resuspended in 6 mL prewarmed medium and continued to be cultured as described above. Transfection efficiency as well as viability was determined by transfecting the cells with 400 nM green fluorescence siRNA (siGLO from Dharmacon, Chicago, IL, USA) followed by propidium iodide exclusion dye and flow cytometric analysis.

### 2.3. Patch-Clamp Recording

Maternal, Bcl-2-overexpressing, and control vector-transfected Jurkat cells were irradiated (IR) with 0, 5, or 10 Gy 6 MV photons by the use of linear accelerator (LINAC SL25 Philips) at a dose rate of 4 Gy/min at room temperature. Whole-cell currents were evoked by 9–11 voltage pulses (700 ms each) to voltages between −100 (−80) mV and +100 (+80) mV delivered in 20 mV increments. Mean steady state current values were analyzed 2–49 h after IR. The liquid junction potentials between the pipette and the bath solutions were estimated according to [32], and data were corrected for the estimated liquid junction potentials. Applied voltages refer to the cytoplasmic face of the membrane with respect to the extracellular space. Inward currents, defined as flow of positive charge from the extracellular to the cytoplasmic membrane face, are negative currents and depicted as downward deflections of the original current traces.

Cells were superfused at 37°C temperature with NaCl ringer solution (in mM: 125 NaCl, 32 n-2-hydroxyethylpiperazine-n-2-ethanesulfonic acid (HEPES), 5 KCl, 5 d-glucose, 1 MgCl_2_, and 1 CaCl_2_, titrated with NaOH to pH 7.4). Upon GΩ-seal formation and entry into the whole-cell recording mode, cells were recorded with NaCl bath solution (in mM: 140 NaCl, 10 HEPES titrated with NaOH to pH 7.4), KCl bath solution (in mM: 140 KCl, 10 HEPES titrated with KOH to pH 7.4), CaCl_2_ bath solution (in mM: 100 CaCl_2_, 10 HEPES, titrated with Ca(OH)_2_ to pH 7.4), or n-methyl-d-glucamine-Cl (NMDG-Cl) bath solution (in mM: 180 mM n-methyl-d-glucamine titrated with HCl to pH 7.4). The K-d-gluconate/KCl pipette solution contained (in mM) 60 K-d-gluconate, 80 KCl, 5 HEPES, 1 MgCl_2_, 1 K_2_-EGTA, and 1 K_2_-ATP, titrated with KOH to pH 7.4. To activate TRPM2 channels, ADP-ribose (1 *μ*M, Sigma, Taufkirchen, Germany) was added to the pipette solution. To inhibit TRPM2 and IK channels* N*-(*p*-amylcinnamoyl)anthranilic acid (ACA, 0 or 20 *μ*M) and TRAM-34 (0 or 10 *μ*M, both from Sigma, both prepared from a 10 mM stock solution in DMSO) were added to the bath solution, respectively.

### 2.4. Querying the Cancer Genome Atlas (TGCA) Data Sets

Via the cBIOportal Web resource [33, 34], 178 hematopoietic and lymphoid tissue cancer cell lines of the Novartis and Broad Institute Cancer Cell Line Encyclopedia [35] were queried for TRPM2 and Bcl-2 mRNA abundance.

### 2.5. Western Blotting

Irradiated Jurkat cells (0, 5, or 10 Gy, 2–4 h after IR) were lysed in a buffer (containing in mM 50 HEPES, pH 7.5, 150 NaCl, 1 EDTA, 10 sodium pyrophosphate, 10 NaF, 2 Na_3_VO_4_, 1 phenylmethylsulfonyl fluoride (PMSF) additionally containing 1% triton X-100, 5 *μ*g/mL aprotinin, 5 *μ*g/mL leupeptin, and 3 *μ*g/mL pepstatin) and separated by SDS-PAGE under reducing condition. In some experiments, cells were preincubated (0.25 h), irradiated (5 Gy), and postincubated (4 h) in the presence of the TRPM2 channel inhibitor ACA (20 *μ*M). Segregated proteins were electrotransferred onto PVDF membranes (Roth, Karlsruhe, Germany). Blots were blocked in TBS buffer containing 0.05% Tween 20 and 5% nonfat dry milk for 1 h at room temperature. The membrane was incubated overnight at 4°C with the following primary antibodies: rabbit anti-phospho-CaMKII (Thr286) antibody (Cell Signaling #3361, New England Biolabs, Frankfurt, Germany, 1 : 1000), rabbit anti-CaMKII (pan) antibody (Cell Signaling #3362, 1 : 1000), rabbit anti-phospho-cdc25b (Ser187) antibody (Epitomics #T1162, Biomol Hamburg, Germany, 1 : 1000), rabbit anti-cdc25b antibody (Cell Signaling #9525, 1 : 1000), rabbit-anti TRPM2 (Bethyl Laboratories Inc., #A300-414A-2, Montgomery, TX, USA, 1 : 300), or mouse anti-Bcl-2 antibody (Santa Cruz Biotechnology, sc-509, Heidelberg, Germany, 1 : 1000). Equal gel loading was verified by an antibody against *β*-actin (mouse anti-*β*-actin antibody, clone AC-74, Sigma #A2228 1 : 20,000). Antibody binding was detected with a horseradish peroxidase-linked goat anti-rabbit or horse anti-mouse IgG antibody (Cell Signaling #7074 and #7076, resp.; 1 : 1000–1 : 2000 dilution in TBS-Tween/5% milk) incubated for 1 h at room temperature and enhanced chemoluminescence (ECL Western blotting analysis system, GE Healthcare/Amersham-Biosciences, Freiburg, Germany) was detected by film autography.

### 2.6. Fura-2 Ca^2+^ Imaging

Fluorescence measurements were performed using an inverted phase-contrast microscope (Axiovert 100; Zeiss, Oberkochen, Germany). Fluorescence was evoked by a filter wheel (Visitron Systems, Puchheim, Germany) mediated alternative excitation at 340/26 or 387/11 nm (AHF, Analysentechnik, Tübingen, Germany). Excitation and emission light was deflected by a dichroic mirror (409/LP nm beamsplitter, AHF) into the objective (Fluar x40/1.30 oil; Zeiss) and transmitted to the camera (Visitron Systems), respectively. Emitted fluorescence intensity was recorded at 587/35 nm (AHF). Excitation was controlled and data acquired by Metafluor computer software (Universal Imaging, Downingtown, PA, USA). The 340/380 nm fluorescence ratio was used as a measure of cytosolic free Ca^2+^ concentration (_free_[Ca^2+^]_i_). The cells were irradiated (0 or 5 Gy) and loaded with fura-2/AM (2 *μ*M for 30 min at 37°C; Molecular Probes, Goettingen, Germany) in supplemented RPMI medium. _free_[Ca^2+^]_i_ was determined 1.5–5 h after IR at 37°C during superfusion with NaCl ringer (see above), upon Ca^2+^ depletion with Ca^2+^-free NaCl ringer solution (in mM: 125 NaCl, 32 HEPES, 5 KCl, 5 d-glucose, 1 MgCl_2_, and 0.5 EGTA, titrated with NaOH to pH 7.4), and during Ca^2+^ readdition in NaCl ringer solution additionally containing ACA (0 or 20 *μ*M).

### 2.7. Flow Cytometry

To test for mitochondrial production of superoxide anion, Jurkat cells were irradiated (0 or 10 Gy), further cultured for 6 h, harvested, washed, and incubated for 10 min at 37°C in NaCl ringer solution (see above) containing 5 *μ*M of the superoxide anion-sensitive dye MitoSOX (Invitrogen) and 0 or 20 *μ*M ACA, and superoxide anion-sensitive fluorescence was recorded by flow cytometry in fluorescence channel Fl-2 (logarithmic scale, 488 nm excitation and 564–606 nm emission wavelengths). To confirm equal fluorescence dye loading, samples were oxidized (10 mM* tert*-butylhydroperoxide) for 12 min and recorded (data not shown).

To monitor mitochondrial function, Jurkat cells were irradiated (0 or 10 Gy) and further cultured for 6 h. Thereafter, cells were harvested, washed, and incubated for 30 min at 37°C in NaCl ringer solution (see above) containing 25 nM of the inner mitochondrial membrane potential (ΔΨ_*m*_) specific dye tetramethylrhodamine ethyl ester perchlorate (TMRE, Invitrogen) and ΔΨ_*m*_ was analyzed by flow cytometry in fluorescence channel FL-2 (logarithmic scale).

For cell cycle analysis, Jurkat cells were preincubated (0.25 h), irradiated (0, 5 or 10 Gy), and incubated for further 24 h in supplemented RPMI 1640 medium additionally containing either ACA or clotrimazole (Sigma, 0 or 20 *μ*M, each). Cells were permeabilized and stained (0.5 h at room temperature) with unsteril (i.e., not RNase-free) propidium iodide (PI) solution (containing 0.1% Na-citrate, 0.1% triton X-100, 10 *μ*g/mL PI in phosphate-buffered saline, PBS), and the DNA amount was analyzed by flow cytometry (FACS Calibur, Becton Dickinson, Heidelberg, Germany, 488 nm excitation wavelength) in fluorescence channel FL-3 (linear scale, >670 nm emission wavelength). In parallel, cells with degraded DNA were defined by the subG_1_ population of the PI histogram recorded in fluorescence channel FL-2 (logarithmic scale).

For determination of _free_[Ca^2+^]_i_ cells were loaded in NaCl ringer solution (see above) for 0.5 h with fluo-3-AM (2 *μ*M in NaCl ringer, Calbiochem; Bad Soden, Germany) and recorded in fluorescence channels FL-1 (logarithmic scale, 515–545 nm emission wavelengths). As loading control for fluo-3, cells were incubated with the Ca^2+^ ionophore ionomycin (1 *μ*M for 10 min) prior to analysis by flow cytometry. Data were analyzed with the FCS Express 3 software (De Novo Software, Los Angeles, CA, USA).

### 2.8. Statistics

Data are expressed as means ± SE and statistical analysis was made by normal or Welch-corrected two-tailed *t*-test or ANOVA where appropriate using InStat software (GraphPad Software Inc., San Diego, CA, USA).

## 3. Results and Discussion

### 3.1. Modulation of on Channel Activity by Ionizing Radiation

**Figure 1:**
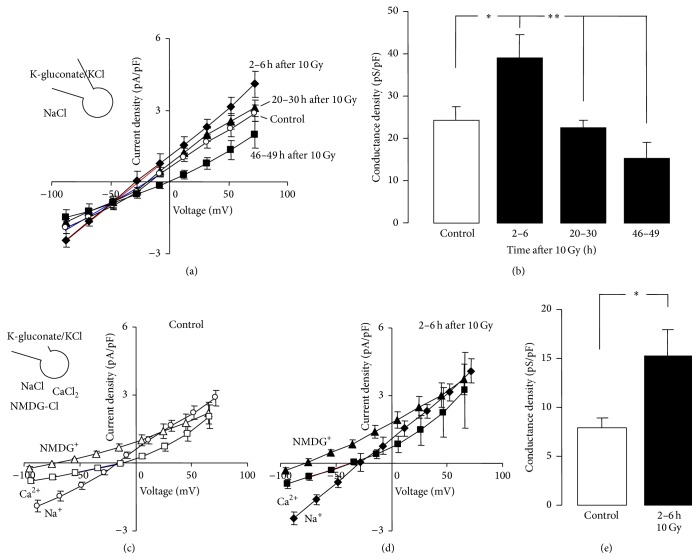
Ionizing radiation (IR) increases the cation and the Ca^2+^ conductance of the plasma membrane in human Jurkat T cell leukemia cells. (a, b) Current density-voltage relationships (*I*/*V* curves, a) and conductance densities (b) of Jurkat cells at different time periods (as indicated) after IR with 0 Gy (control, open circles and bar) or 10 Gy (closed symbols and bars). Currents were recorded in whole-cell voltage-clamp mode with K-gluconate/KCl pipette and NaCl bath solution and elicited by 9 voltage square pulses to voltages between −80 mV and +80 mV (20 mV increments). Conductance densities were calculated for the inward currents as shown by the blue and red line in (a) for control cells and irradiated cells (2–6 h after IR), respectively. (c, d)* I*/*V* curves of control (c) and irradiated Jurkat cells (2–6 h after 10 Gy, d) recorded as in (a) with NaCl bath solutions (circles) or after replacement of Na^+^ with Ca^2+^ (squares) or the impermeable cation n-methyl-d-glucamine (NMDG, triangles). (e) Ca^2+^ conductance density of control cells (open bar) and irradiated Jurkat cells (2–6 h after 10 Gy, closed bar). The blue and red line in (c) and (d), respectively, show the voltage range used for calculation of the Ca^2+^ conductance densities. Data are means ± SE, *n* = 5 for the 46–49 h values in (a) and *n* = 8–15 for all other data. *∗* and *∗∗* indicate *p* ≤ 0.05 and 0.01 as tested by ANOVA (b) and Welch-corrected *t*-test (e), respectively.

To assess the effect of ionizing radiation (IR) of ion channel activity, Jurkat cells were irradiated with 10 Gy and whole-cell currents were recorded at different time periods after IR. As shown in Figures 1(a) and 1(b), IR induced an increase in whole-cell currents 2–6 h after IR. Substitution of Na^+^ in the bathing solution by Ca^2+^ or the impermeable Na^+^ substitute NMDG^+^ indicated both cation-selectivity and Ca^2+^ permeability of the IR-induced currents (Figures 1(c)–1(e)).

**Figure 2:**
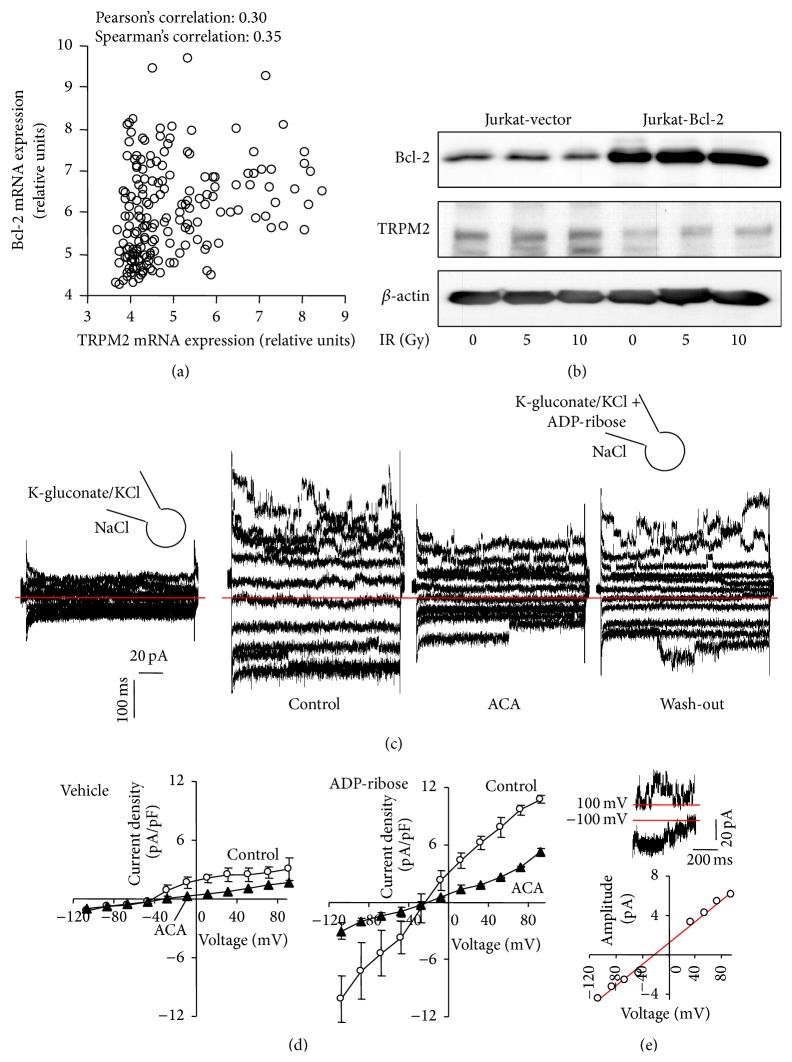
T cell leukemia cells functionally express TRPM2 Ca^2+^-permeable cation channels and TRPM2 expression correlates with that of the antiapoptotic protein Bcl-2. (a) Dot blot showing the relative mRNA abundances of TRPM2 and Bcl-2 in 178 hematopoietic and lymphoid tissue cancer cell lines. Data are from the Novartis and Broad Institute Cancer Cell Line Encyclopedia. (b) Immunoblots from whole lysates of irradiated (0, 5, or 10 Gy, 4 h after IR) stably transfected control (Jurkat-vector) and Bcl-2-overexpressing (Jurkat-Bcl-2) cells probed against Bcl-2, TRPM-2, and *β*-actin. (c) Current tracings recorded as in Figure 1(a) in Jurkat-Bcl-2 cells with vehicle alone (1st tracings) or in an unpaired experiment with the TRPM-2 activator ADP-ribose (1 *μ*M) in the pipette (2nd–4th tracings). The recordings with ADP-ribose were performed before (2nd tracings, control), during (3rd tracings, ACA), and after (4th tracings, wash-out) bath application of the TRPM-2 inhibitor n-(p-amylcinnamoyl)-anthranilic acid (ACA, 20 *μ*M, zero currents are shown by red lines). (d)* I*/*V* curves of the mean whole cell currents (± SE, *n* = 3) of Jurkat-Bcl-2 cells recorded the absence (left) or presence of the TRPM2-activator ADP-ribose (right) in the pipette before (open circles) and after bath superfusion with the TRPM2 inhibitor ACA (closed triangles). (e) Single channel characteristics of the ADP-ribose-stimulated channel. Unitary current transitions were apparent in whole-cell currents tracings as depicted here for −100 mV and +100 mV clamp-voltage in the upper panel. The lower panel shows the relationship between unitary current transitions and voltage indicating a unitary conductance of about 50 pS.

Next, the functional expression of TRPM2 channels and its dependence on Bcl-2 was determined in Jurkat cells. Such dependence was suggested by a positive correlation of the TRPM2 and Bcl-2 mRNA abundances in 178 hematopoietic and lymphoid tissue cancer cell lines of the Novartis and Broad Institute Cancer Cell Line Encyclopedia (Figure 2(a)). In the Jurkat cell model, in contrast, TRPM2 protein abundance seemed to be lower in Bcl-2-overexpressing (Jurkat-Bcl-2) cells as in the control vector-transfected (Jurkat-vector) cells as suggested by immunoblotting (Figure 2(b)). IR did not modify total TRPM2 protein content of the cells (Figure 2(b)).

To activate TRPM2 in Jurkat cells, whole-cell currents were recorded with the TRPM2 agonist ADP-ribose in the pipette and compared in unpaired experiments with those recorded under control conditions. Intracellular ADP-ribose stimulated a whole-cell current fraction which was sensitive to the unspecific TRPM2 inhibitor ACA [36] (Figures 2(c) and 2(d)). Importantly, ADP-ribose-stimulated currents exhibited unitary current transitions with a unitary conductance of some 50 pS as reported for heterologously expressed TRPM2 channels [37] (Figure 2(e)). Together, these data indicated functional expression of TRPM2 in Jurkat cells.

### 3.2. Mitochondrial Superoxide Anion Formation: Effect of Ionizing Radiation, Bcl-2 Overexpression, and TRPM2 Inhibition

**Figure 3:**
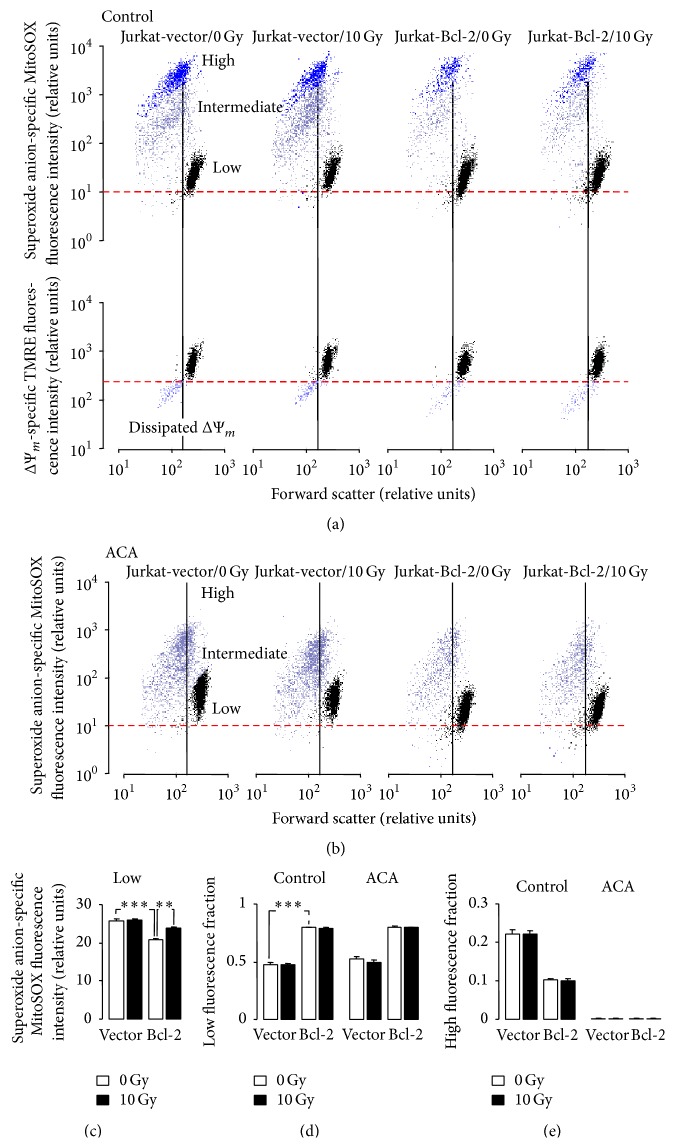
IR increases mitochondrial superoxide anion formation in Bcl-2-overexpressing cells. (a, b) Dot plots showing forward scatter and the superoxide anion-selective MitoSOX fluorescence (a, upper panel, and b) as well as the inner mitochondrial membrane potential ΔΨ_*m*_-specific TMRE fluorescence (a, lower panel) of Jurkat-vector- (1st and 2nd panels) and Jurkat-Bcl-2 cells (3rd and 4th panels) 6 h after irradiation with 0 Gy (1st and 3rd panels) or 10 Gy (2nd and 4th panels). Incubation (10 min at 37°C) with the superoxide anion-sensitive fluorescence dye was carried out in the absence (a) or presence (b) of the TRPM2 inhibitor ACA (20 *μ*M). Three distinct cell populations with low (black), intermediate (lilac), or high (blue) superoxide anion formation were apparent. The majority of intermediate and high superoxide anion-forming cells exhibited a low forward scatter which was associated with dissipation of ΔΨ_*m*_ (a, lower panel). (c) Mean (± SE, *n* = 4) MitoSOX fluorescence intensity in the low-fluorescent populations of 0 Gy- (open bars) or 10 Gy-irradiated (closed bars, 6 h after irradiation) Jurkat-vector- (left) and Jurkat-Bcl-2 cells (right). (d, e) Mean (± SE, *n* = 4) fraction of MitoSOX low-fluorescent (d) and high-fluorescent Jurkat-vector- (1st, 2nd, 5th, and 6th bars) and Jurkat-Bcl-2 cells (3rd, 4th, 7th, and 8th bars) 6 h after irradiation with 0 Gy (open bars) or 10 Gy (closed bars). The incubation with the fluorescence dye was carried out in the absence (1st–4th bars) or presence (5th–8th bars) of ACA (20 *μ*M). *∗∗* and *∗∗∗* indicate *p* ≤ 0.01 and *p* ≤ 0.001, respectively, ANOVA.

To assess IR-stimulated formation of superoxide anion by mitochondria and to estimate a potential role of TRPM2 channels herein, Jurkat-Bcl-2 and Jurkat-vector cells were irradiated (0 or 10 Gy), postcultured for 6 h, and incubated for 10 min with the superoxide anion-sensitive fluorescence dye MitoSOX. The dye incubation was performed in the absence or presence of the TRPM2 inhibitor ACA. As shown in Figure 3(a), upper panel, and Figure 3(b), three distinct cell populations with low, intermediate, or high MitoSOX fluorescence were apparent. The latter two showed lower cell sizes as compared to the low-fluorescent population, suggestive of superoxide anion formation-associated cell shrinkage. Staining of the cells in parallel experiments with the inner mitochondrial membrane potential (ΔΨ_*m*_) specific dye TMRE indicated dissipation of ΔΨ_*m*_ in most of the shrunken cells (Figure 3(a), lower panel) suggesting that the vast majority of cells with intermediate and high MitoSOX fluorescence underwent apoptotic cell death.

The low-fluorescent, nonshrunken cell population was larger and exhibited significant lower superoxide anion formation in Jurkat-Bcl-2 cells as compared to this population in Jurkat-vector cells (open bars in Figures 3(c) and 3(d), left). In the low-fluorescent populations, irradiation significantly increased the superoxide formation only in Jurkat-Bcl-2 cells to levels which still remained below those of control or irradiated Jurkat-vector cells (compare open and closed bars in Figure 3(c)). Importantly, ACA slightly but significantly (*p* ≤ 0.05, ANOVA) decreased superoxide anion formation in unirradiated (from 20.9 ± 0.21 to 18.2 ± 0.22 relative units, *n* = 4) and irradiated (from 23.8 ± 0.26 to 21.6 ± 0.79 rel. units, *n* = 4) low-fluorescent Jurkat-Bcl-2 cells while having no inhibiting effect on superoxide anion formation in low-fluorescent Jurkat-vector cells (data not shown). IR effects on the intermediate- or high-fluorescent populations of both cell clones, in contrast, were not apparent (Figure 3(a)). ACA markedly decreased the superoxide formation of the intermediate- or high-fluorescent populations in all control or irradiated cells (compare Figures 3(a) and 3(b)) resulting in the disappearance of the high-fluorescent cells (Figure 3(e)).

Combined, these data demonstrate lower mitochondrial superoxide anion formation in Jurkat-Bcl-2 cells as compared to Jurkat-vector cells. Only in the former cells, IR induced an increase in superoxide anion formation. In addition, superoxide anion formation was lowered by the TRPM2 inhibitor ACA in cells of both clones independent of IR stress. This might suggest a contribution of TRPM2-mediated Ca^2+^ uptake to mitochondrial ROS formation.

### 3.3. Ionizing Radiation-Stimulated Ca^2+^ Uptake: Regulation by Bcl-2 and Involvement of TRPM2 Channels

**Figure 4:**
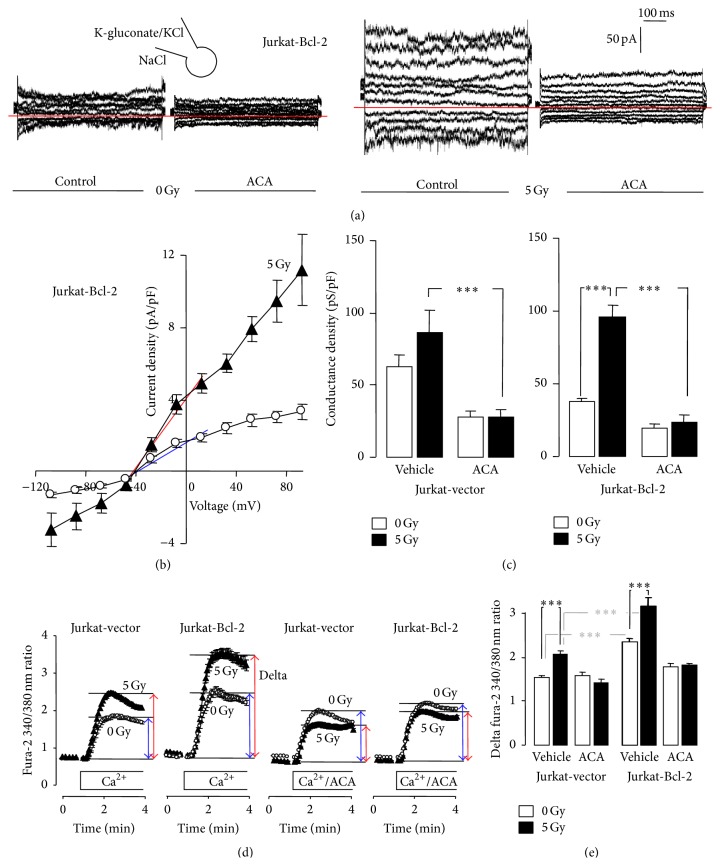
IR stimulates Ca^2+^ entry through TRPM2 channels especially in Bcl-2-overexpressing Jurkat cells. (a) Whole-cell current tracings recorded in Jurkat-Bcl-2 cells irradiated with 0 Gy (1st and 2nd tracings) or 5 Gy (3rd and 4th tracings, 2 h after IR). Records were obtained in unpaired experiments as described in Figure 1(a) before (1st and 3rd tracings) and during bath application of the TRPM2 inhibitor ACA (20 *μ*M, 2nd and 4th tracings). (b) Relationship of the mean (± SE, *n* = 7–10) current density and the voltage recorded as in (a) in Jurkat-Bcl-2 cells irradiated with 0 Gy (open circles) or 5 Gy (closed triangles). (c) Mean (± SE, *n* = 6–12) conductance density of control (0 Gy, open bars) and irradiated (5 Gy, 2–5 h after IR) Jurkat-vector (left) and Jurkat-Bcl-2 cells (right) recorded as in (a) in the absence or presence of ACA. Conductance densities were calculated for the outward currents as shown by the blue and red line in (b) for control and irradiated cells, respectively. (d, e) Mean (± SE, *n* = 197–336) fura-2 340/380 nm ratio (d) and delta fura-2 ratio (e) as measures of cytosolic free Ca^2+^ concentration and Ca^2+^ entry in Ca^2+^-depleted cells, respectively. Ca^2+^-specific fura-2 fluorescence was recorded by imaging in control (0 Gy, open circles and bars) and irradiated (5 Gy, closed triangles and bars, 1.5–5 h after IR) Jurkat-vector and Jurkat-Bcl-2 cells using extracellular Ca^2+^ removal/readdition protocol. Ca^2+^ readdition was performed in the absence (vehicle) or presence of ACA (20 *μ*M). *∗∗∗* indicates *p* ≤ 0.001, ANOVA.

To determine IR-induced changes in TRPM2 activity, irradiated (0 or 5 Gy) Jurkat-Bcl-2 and Jurkat-vector cells were whole-cell recorded in the absence and presence of ACA (Figures 4(a)–4(c)). The ACA-sensitive current fraction of nonirradiated cells was higher in Jurkat-vector than in Jurkat-Bcl-2 cells (compare 1st with 3rd and 5th with 7th bar in Figure 4(c), resp.) which might reflect the observed differences in TRPM2 protein abundance and which is in accordance with the observed differences in mitochondrial ROS formation. IR (5 Gy) stimulated an increase in the ACA-sensitive currents predominately in Jurkat-Bcl-2 cells (Figure 4(c)) which again might be mirrored by the observed IR sensitivity of mitochondrial ROS formation exclusively in Jurkat-Bcl-2 cells.

In accordance with an IR-induced increase in TRPM2 activity, IR stimulated an ACA-sensitive Ca^2+^ uptake as measured by fura-2 Ca^2+^ imaging using an extracellular Ca^2+^ depletion/repletion protocol (Figure 4(d)). In contrast to the ACA-sensitive basal whole cell currents (Figure 4(b)), basal (ACA-sensitive) Ca^2+^ uptake was higher in Jurkat-Bcl-2 than in Jurkat-vector cells (compare 1st with 5th bar in Figure 4(e)). Similarly to the whole-cell currents, IR (5 Gy) stimulated a larger Ca^2+^ uptake in Jurkat-Bcl-2 as compared to Jurkat-vector cells (compare 2nd with 6th bar in Figure 4(e)). In the presence of ACA, Ca^2+^ uptake did not differ between control and irradiated Jurkat-vector and Jurkat-Bcl-2 cells (3rd, 4th, 7th, and 8th bar in Figure 4(e)). Together, these observations indicated an IR-stimulated Bcl-2-regulated Ca^2+^ uptake in Jurkat cells which probably involves TRPM2 channels.

### 3.4. Role of TRPM2 Channels in Ionizing Radiation-Stimulated Activation of Ca^2+^ Effector Proteins Involved in Cell Cycle Arrest

**Figure 5:**
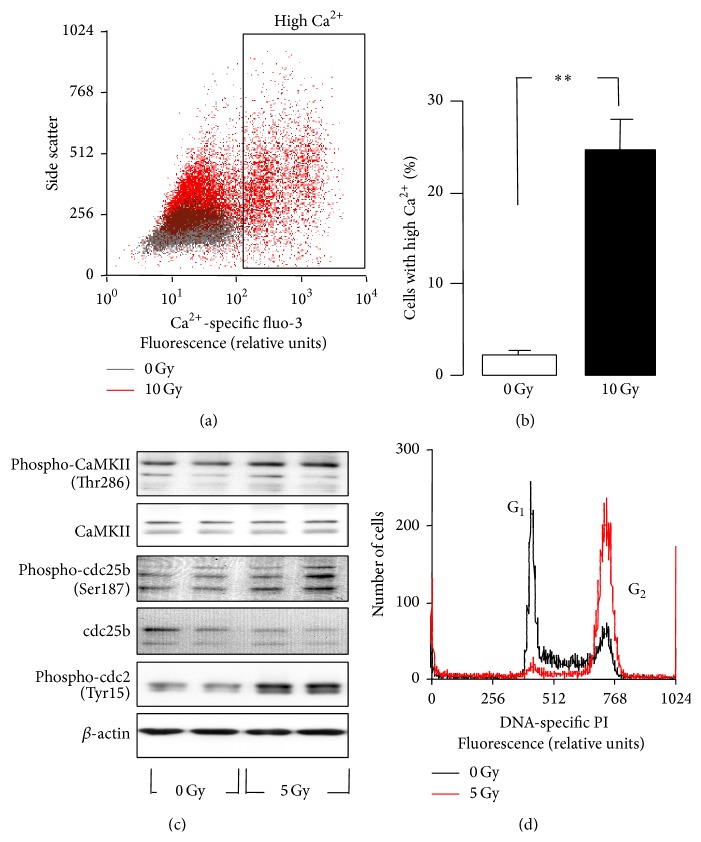
IR induces Ca^2+^ signaling and a G_2_/M cell cycle arrest in Jurkat cells. (a) Dot plot recorded by flow cytometry showing the Ca^2+^-specific fluo-3 fluorescence intensity in dependence on side scatter of Jurkat cells 24 h after IR with 0 Gy (grey) or 10 Gy (red). (b) Mean (± SE, *n* = 4) percentage of control and irradiated Jurkat cells with high cytosolic free Ca^2+^ concentrations (determined by fluo-3 fluorescence in flow cytometry as described in (a), *∗∗* indicates *p* ≤ 0.01, Welch-corrected *t*-test). (c) Immunoblots from whole lysates of irradiated (0 or 5 Gy, 4 h after IR) Jurkat cells probed against phosphorylated and total Ca^2+^/CaM-dependent kinase II (CaMKII) isoforms, against the phosphorylated and total phosphatase cdc25b, the phosphorylated cell division cycle protein 2 (cdc2), and *β*-actin for loading control. (d) Flow cytometry histogram depicting the fluorescence intensity of the DNA-specific dye propidium iodide (PI) in Jurkat cells 24 h after IR with 0 (black) or 5 Gy (red).

This IR-stimulated Ca^2+^ uptake might be hazardous for the cells leading to Ca^2+^ overflow and subsequent cell death. In fact, 24 h after IR with 10 Gy, some 25% of the Jurkat cells exhibited a highly increased _free_[Ca^2+^]_i_ as deduced from fluo-3 flow cytometry (Figures 5(a) and 5(b)). Ca^2+^ uptake might also contribute to Ca^2+^ signaling that is required for DNA damage response of the irradiated T cell leukemia cells. IR (5 Gy) stimulated autophosphorylation and activation of Ca^2+^/calmodulin-dependent protein kinase II (CaMKII) isoforms and phosphorylation-dependent inactivation of the CaMKII downstream target cdc25b as suggested by immunoblotting (Figure 5(c)). Inactivation of the phosphatase cdc25b was parallel by radiation-induced phosphorylation-dependent inactivation of the cdc25b substrate cdc2 (Figure 5(c)). This might hint to an involvement of Ca^2+^ effector proteins such as CaMKII in G_2_/M arrest as observed in PI flow cytometry 24 h after IR (5 Gy, Figure 5(d)).

**Figure 6:**
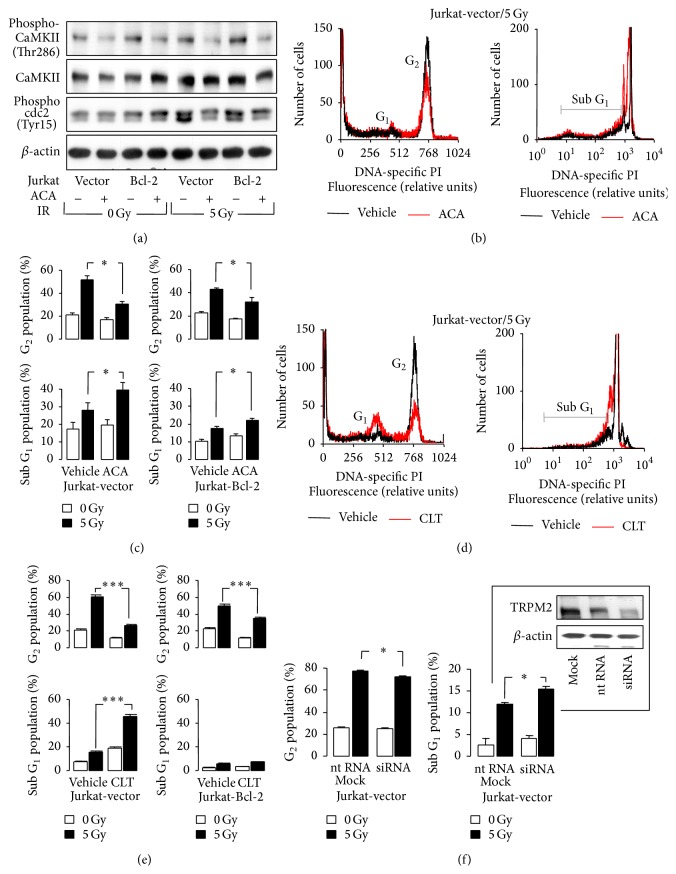
Ca^2+^-signaling via ACA-sensitive Ca^2+^ entry contributes to IR-induced G_2_/M cell cycle arrest and decreases IR-induced cell death of Jurkat cells. (a) Immunoblots from whole lysates of irradiated (0 or 5 Gy, 4 h after IR) Jurkat-vector and Jurkat-Bcl-2 cells probed against phospho-CaMKII and total CaMKII isoforms, against phospho-cdc2, and against *β*-actin. Cells were irradiated and postincubated in the presence of ACA (0 or 20 *μ*M). (b, d) Histograms showing the DNA-specific PI fluorescence intensity of irradiated (5 Gy, 24 h after IR) Jurkat-vector cells pre- (0.25 h) and postincubated (24 h) with 0 *μ*M (black) or 20 *μ*M ACA (red) in (b) or 0 *μ*M (black) or 20 *μ*M clotrimazole (CLT, red) in (d). (c, e) Mean (± SE, *n* = 9–12) percentage of irradiated (0 Gy, open bars, or 5 Gy, closed bars) and ACA- in (d) or CLZ- in (e) (both 0 or 20 *μ*M) cotreated Jurkat-vector and Jurkat-Bcl-2 cells arrested in G_2_ phase of cell cycle (upper line) or belonging to the dead cells accumulating in the subG_1_ population (lower line). (f) Knock-down of TRPM2 by RNA interference mimics the effect of ACA. Electroporation with TRPM2-specific siRNA decreases the TRPM2 protein abundance in Jurkat-vector cells to about a half of that of nontargeting RNA- (nt RNA-) transfected control cells as analyzed by TRPM2 and *β*-actin immunoblots, 48 h after electroporation (*insert*, mock: electroporation without RNA). Lower line: mean (± SE, *n* = 3–6) percentage of irradiated (0 Gy, open bars, or 5 Gy, closed bars, 24 h after IR) in G_2_/M arrest (left) or in subG_1_ population as analyzed by PI staining and flow cytometry as shown in (b, d). Cells were either mock-electroporated or transfected with nt RNA or TRPM2-specific siRNA. Mock and nt RNA data did not differ and were pooled. *∗* and *∗∗∗* indicate *p* ≤ 0.05 and *p* ≤ 0.001, ANOVA, respectively.

To confirm an involvement of TRPM2 and CaMKII in the stress response of Jurkat cells, Jurkat-Bcl-2 and Jurkat-vector cells were irradiated (0 or 5 Gy) and postincubated in the presence or absence of the TRPM2 inhibitors ACA or clotrimazole [38, 39] and kinase activities of the CaMKII isoforms and cdc2 and cell cycle distribution and cell death were analyzed by immunoblotting and PI flow cytometry, 4 h and 24 after IR, respectively. ACA decreased the basal and radiation-induced abundance of phosphorylated CaMKII in Jurkat-Bcl-2 and Jurkat-vector cells (Figure 6(a), 1st and 2nd blot). Most importantly, ACA blocked the radiation-induced phosphorylation-dependent inactivation of cdc2 in both genotypes (Figure 6(a), 3rd blot) suggesting a functional significance of ACA-sensitive Ca^2+^ entry for G_2_/M cell cycle arrest. Accordingly, ACA and clotrimazole decreased the number of irradiated cells arrested in G_2_/M (Figures 6(b)–6(e)) and increased the number of dead cells. ACA- and clotrimazole-induced cell death was more pronounced in irradiated Jurkat-vector than in Jurkat-Bcl-2 cells (subG_1_ population, Figures 6(b)–6(e)).

Finally, the function of TRPM2 in radiation-induced G_2_/M arrest of Jurkat cells was directly tested by TRPM2 knock-down. Transfection of Jurkat-vector cells with TRPM2 siRNA resulted in downregulation of TRPM2 protein level as compared to nontargeting (nt) RNA-transfected cells (Figure 6(f), insert). Transfected Jurkat cells were irradiated (0 or 5 Gy) and G_2_/M arrest and cell death analyzed 24 h thereafter. TRPM2 knock-down exerted small but significant effects on radiation-induced G_2_/M arrest and cell death mimicking those of ACA and clotrimazole (Figure 6(f)).

### 3.5. Regulation of TRPM2-Mediated Ca^2+^ Influx by Mitochondria and Bcl-2

ADP-ribose is liberated in the mitochondria from, for example, NAD-dependent deacetylation intermediates, from mono- or polyADP-ribosylated proteins, or from NAD^+^, and released into the cytosol [19]. Oxidative and nitrosative stress have been demonstrated to stimulate the mitochondrial release of ADP-ribose into the cytosol which in turn activates TRPM2 channels in the plasma membrane resulting in Ca^2+^ entry and depolarization of the membrane potential [16]. Since an elevated _free_[Ca^2+^]_i_ may disinhibit the respiration change leading to ΔΨ_*m*_ hyperpolarisation and superoxide anion formation and, eventually, to mitochondrial Ca^2+^ overload and ΔΨ_*m*_ dissipation, TRPM2 activation has been proposed to amplify signals that trigger cell death (for review see [27]).

The present study demonstrates that irradiated human T cell leukemia cells may utilize the TRPM2 “death channel” for prosurvival Ca^2+^ signaling. Noteworthy, IR-induced TRPM2 currents and Ca^2+^ entry were larger in cells overexpressing Bcl-2 pointing to a crosstalk between Bcl-2 in the ER and outer mitochondrial membrane and TRPM2 in the plasma membrane. The correlation between TRPM2 and Bcl-2 mRNA abundances in a panel of lymphohematopoietic cancer cell lines (see Figure 2(a)) further suggests a functional interdependence between both proteins.

In some cell models, Bcl-2-overexpressing cells have been proposed to counteract the Bcl-2-mediated Ca^2+^ leakage from the stores by downregulating Ca^2+^ uptake through the plasma membrane (for review see [19]). In line with such compensatory mechanism might be the observation of the present study that Bcl-2-overexpressing Jurkat cells exhibited under basal conditions lower TRPM2 protein abundance, smaller ACA-sensitive currents in patch-clamp whole-cell recordings than the control vector-transfected cells (see Figure 2(b)).

In intact cells (i.e., in fura-2 Ca^2+^ imaging experiments, see Figure 4(e)), however, a basal ACA-sensitive Ca^2+^ uptake fraction was only apparent in Bcl-2-overexpressing cells suggestive of a TRPM2 inactivity in control cells under resting conditions. Compared to control cells, the more sustained Ca^2+^ uptake in Bcl-2-overexpressing cells (see Figures 4(d) and 4(e)) suggests that Bcl-2 overexpression might be associated with a set-point shift of the resting _free_[Ca^2+^]_i_ towards higher levels. Fura-2 Ca^2+^ imaging and fluo-3 flow cytometry recordings of the present study indeed demonstrated a higher basal _free_[Ca^2+^]_i_ in constitutively and inducibly Bcl-2-overexpressing Jurkat cells as compared to the respective control cells (see Supplementary Figure A, in Supplementary Material available online at http://dx.doi.org/10.1155/2016/8026702). Elevated _free_[Ca^2+^]_i_ levels reportedly facilitate TRPM2 activation by ADP-ribose [40]. The observed basal ACA-sensitive Ca^2+^ uptake that occurred exclusively in Bcl-2-overexpressing cells might, therefore, be simply explained by a higher basal _free_[Ca^2+^]_i_ in Bcl-2-overexpressing as compared to control cells.

Noteworthy, despite higher basal _free_[Ca^2+^]_i_, Bcl-2-overexpressing cells exhibited lower basal mitochondrial ROS formation than control cells (see Figure 3(c)) suggestive of a Bcl-2-mediated protection of mitochondrial superoxide anion formation. As a matter of fact, a direct promoting function of mitochondrial superoxide anion formation has been attributed to the Bcl-2 opponent Bax in neuronal cells [41].

### 3.6. Rearrangements of the Ca^2+^ Signalosome in Tumor Cells: Functional Significance for Cell Cycle Control and Stress Response

In many tumor entities rearrangements of the Ca^2+^ signalosome have been reported. In prostate cancer, for instance, malignant progression is reportedly accompanied by TRPM8-mediated Ca^2+^ store depletion and downregulation of store-dependent Ca^2+^ entry across the plasma membrane. In exchange, TRP channels such as TRPV6 are upregulated in the plasma membrane of advanced prostate cancer cells which have been proposed to generate in concert with IK K^+^ channels survival and growth factor-independent Ca^2+^ signaling (for review see [42]).

In the present study, IR stimulated the ACA-sensitive currents of Jurkat cells in patch-clamp recordings and the ACA-sensitive Ca^2+^ uptake in fura-2 imaging experiments suggesting an IR-induced increase in TRPM2 activity. IR-induced modifications of ion channel activity have been reported in different tumor entities where they contribute to stress evasion [43], glucose fueling [44, 45], cell cycle control [46, 47], or radioresistance [48].

The p53-mutated Jurkat cells [49] accumulate in G_2_M cell cycle arrest upon IR-mediated DNA damage (see Figure 5(d)). The proposed IR-stimulated Ca^2+^ entry through TRPM2 channels most probably contributed to the G_2_M cell cycle arrest. This was evident from the observation of the present study that two nonspecific TRPM2 inhibitors or TRPM2 knock-down decreased the number of cells accumulating in G_2_ and increased the number of dead cells (see Figure 6). One might speculate that TRPM2 inhibition or knock-down overrides G_2_M cell cycle arrest and forces cells with unrepaired DNA damage into mitosis.

This scenario is strengthened by the observation that IR promoted the _free_[Ca^2+^]_i_-dependent phosphorylation of CaMKIIs and their downstream targets cdc25b and cdc2 in an ACA-sensitive manner (see Figures 5(c) and 6(a)). CaMKIIs phosphorylate and thereby inactivate the phosphatase cdc25b which results in accumulation of the phosphorylated, inactive form of the mitosis promoting factor subunit cdc2 [47]. Combined, these observations suggest that IR-dependent TRPM2 activation contributes to Ca^2+^ signals that are able to induce autophosphorylation and thereby activation of CaMKIIs.

Likewise, irradiated human myeloid leukemia cells have been shown to generate Ca^2+^ signals by the concerted action of TRPV5/6 and Kv3.4 K^+^ channels in the plasma membrane. These Ca^2+^ signals program G_2_M cell cycle arrest similarly to proposed mechanism of the present study via CaMKIIs, cdc25b, and cdc2 [46, 47]. K^+^ channel activity in close vicinity to Ca^2+^ entry pathways maintains a high inwardly directed driving force for Ca^2+^ and, thus, is indispensable for robust Ca^2+^ signals. In analogy to the leukemia cells [47], IR induced the coactivation of IK K^+^ channels in the plasma membrane of Jurkat cells (see supplementary Figure B). This points to both a common signaling in irradiated myeloid and lymphoblastic leukemia cells and the possibility that functionally equivalent Ca^2+^ signals can be generated during DNA damage response by different sets of TRP and K^+^ channels in the plasma membrane.

### 3.7. Conclusions

Plasma membrane TRPM2 channels have been attributed tumor suppressor function in several tumor entities. The Ca^2+^ signalosome of human T cell leukemia cells comprises TRPM2 channels that are activated during DNA damage response. In particular, irradiated Jurkat cells utilize TRPM2 to control the G_2_/M cell cycle arrest probably via activation of the Ca^2+^ effector protein CaMKII and subsequent inhibition of cdc25b and cdc2. The antiapoptotic protein Bcl-2 in the ER or outer mitochondrial membrane even fosters TRPM2 activity presumably by inducing higher _free_[Ca^2+^]_i_ levels and decreases at the same time mitochondrial ROS formation. By doing so, Bcl-2-overexpressing cells may harness TRPM2-generated Ca^2+^ signals without running into the risk of hazardous mitochondrial ROS formation. Thus, Bcl-2 function on mitochondrial integrity and stress-induced TRPM2-mediated Ca^2+^ signaling cooperate in resistance to radiation therapy in T cell leukemia cells.

## Supplementary Material

Overexpression of the anti-apoptotic protein Bcl-2 in Jurkat T cell leukemia cells is associated with an elevated basal cytosolic free Ca^2+^ concentration (Suppl. Figure A) and an increased activity of Ca^2+^-activated IK K^+^ channels especially during stress response upon irradiation. (Suppl. Figure B).
