# Supplementary figures and images for: Targeting TRPM2 Channels Impairs Radiation-Induced Cell Cycle Arrest and Fosters Cell Death of T Cell Leukemia Cells in a Bcl-2-Dependent Manner
Source: Oxid Med Cell Longev. 2015 Dec 29;2016:8026702. doi: 10.1155/2016/8026702 (PMC4709732; doi:10.1155/2016/8026702)

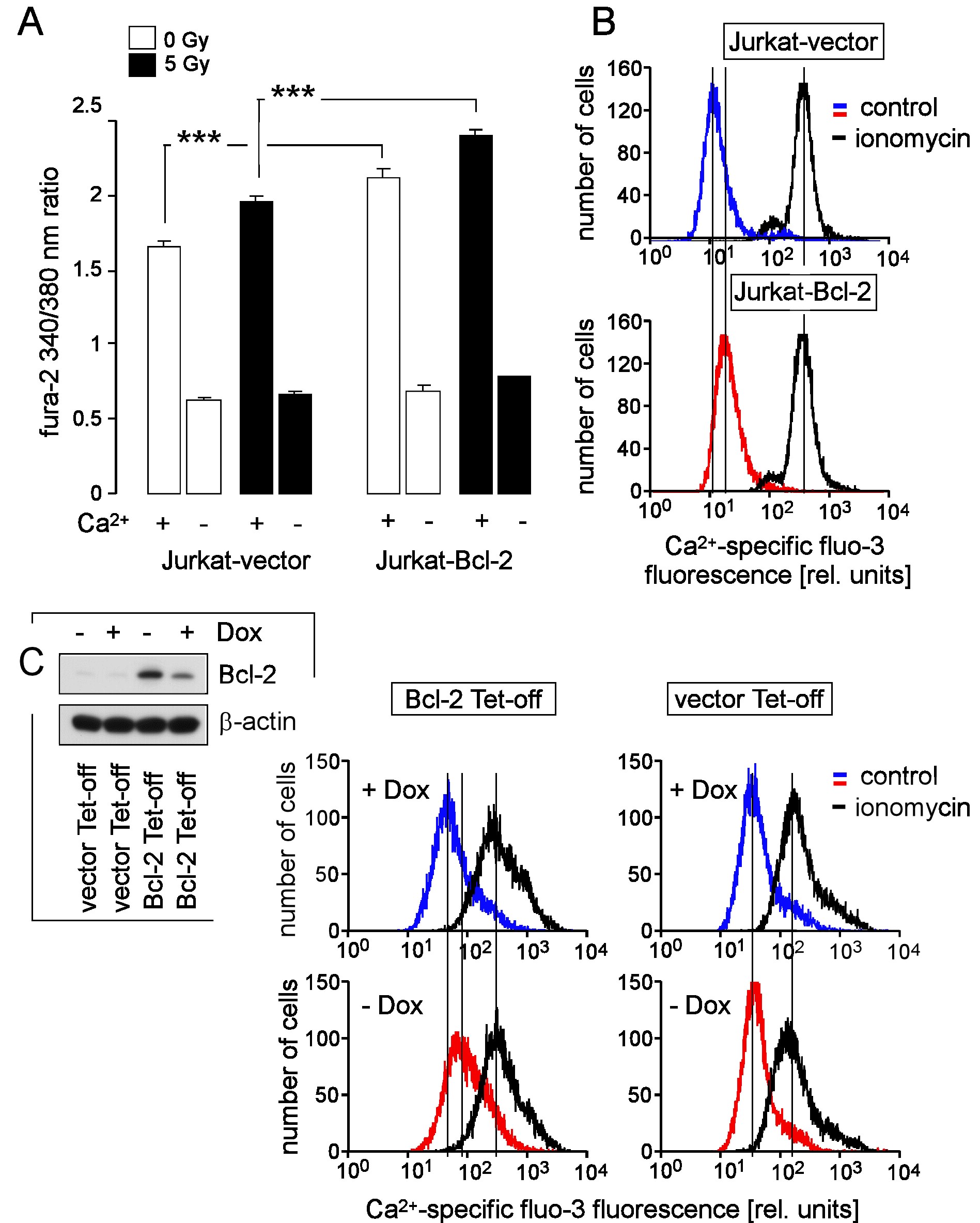

Supplement: Supplementary file 1 — Overexpression of the anti-apoptotic protein Bcl-2 in Jurkat T cell leukemia cells is associated with an elevated basal cytosolic free Ca2+ concentration (Suppl. Figure A) and an increased activity of Ca2+-activated IK K+ channels especially during stress response upon irradiation. (Suppl. Figure B). [file 8026702.f1.zip › 8026702.f1/Suppl_FigA_OXIMED_1470295.jpg]

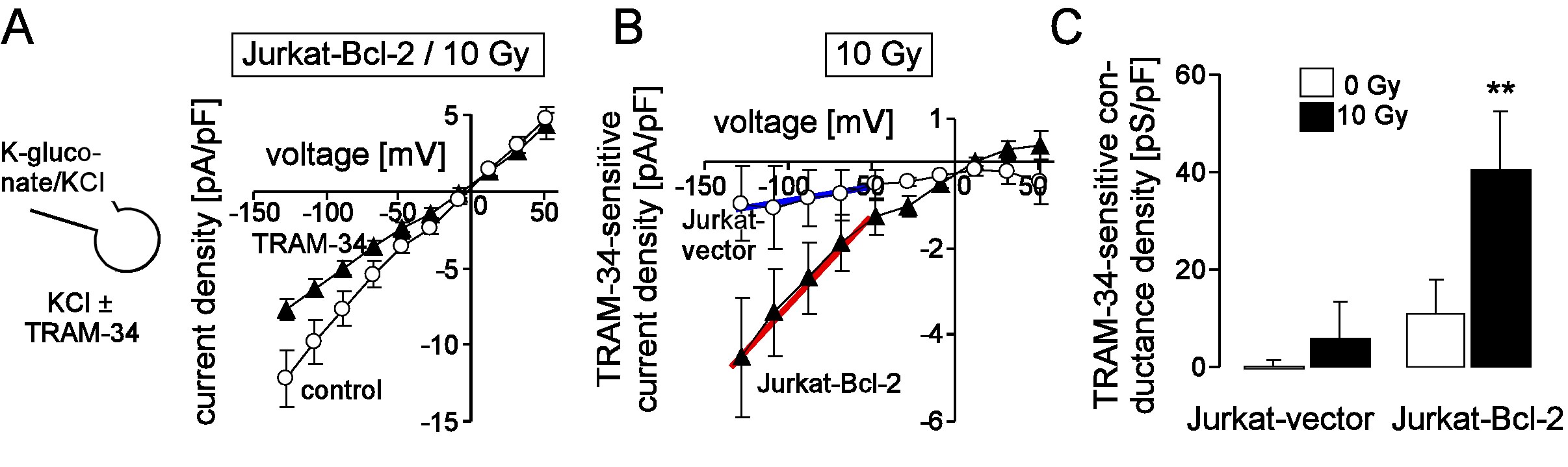

Supplement: Supplementary file 1 — Overexpression of the anti-apoptotic protein Bcl-2 in Jurkat T cell leukemia cells is associated with an elevated basal cytosolic free Ca2+ concentration (Suppl. Figure A) and an increased activity of Ca2+-activated IK K+ channels especially during stress response upon irradiation. (Suppl. Figure B). [file 8026702.f1.zip › 8026702.f1/Suppl_FigB_OXIMED_1470296.jpg]
